# Silent Entry: The Diagnostic Challenge of Tetanus Without an Obvious Wound

**DOI:** 10.7759/cureus.71432

**Published:** 2024-10-14

**Authors:** Kajananan Sivagurunathan, Prashanthan Kaneshamoorthy, Anoja Mathievaanan, Nalayini Jegathesan, Peranantharajah Thampipillai

**Affiliations:** 1 Internal Medicine, Teaching Hospital Jaffna, Jaffna, LKA

**Keywords:** clostridium tetani, spatula test, tetanus, tetanus immune globulin, woundless tetanus

## Abstract

Tetanus, caused by *Clostridium tetani*, is a significant health problem, particularly in regions lacking proper vaccination coverage against tetanus. Although it is usually associated with an identifiable wound, diagnosing tetanus without a visible entry site can be difficult. We report a 68-year-old man who was diagnosed with tetanus without any visible wounds or recent trauma. He had classic clinical features, including trismus, risus sardonicus, muscle spasms, and a positive spatula test, which all justified the clinical diagnosis of tetanus. The patient was treated with intramuscular injection of 3,000 IU of tetanus immune globulin, intravenous metronidazole, and muscle relaxants. He improved significantly after 10 days. He was discharged with follow-up and vaccination instructions. This case emphasizes the importance of maintaining a high index of suspicion for tetanus, even without any wound. It also reveals the effectiveness of clinical diagnostic methods and appropriate, timely treatment in rescuing the patient.

## Introduction

Tetanus is a life-threatening condition caused by *Clostridium tetani*, which belongs to the group of spore-forming anaerobic bacteria. It is found in soil, dust, and animal feces. Tetanus is a significant public health problem, particularly in low- and middle-income countries with incomplete vaccination coverage [1]. From 1990 to 2019, global tetanus cases dropped by 88%, falling from 615,728 to 73,662. During the same period, global tetanus mortality has significantly decreased by 87%, decreasing from 275,379 in 1990 to 34,684 in 2019 [2]. This shows the positive outcomes of vaccination and prevention efforts. In 2023, global DTP3 vaccine coverage for one-year-olds reached 84%, a slight recovery from the decline caused by the COVID-19 pandemic, which reduced coverage from 86% in 2019 to 81% in 2021. Despite these gains, 14.5 million infants are still not vaccinated, and another 6.5 million have only received partial immunization [3]. Risk factors of tetanus include missing vaccines, infected wounds with necrotic tissues, and exposure to contaminated environments. Individuals with chronic skin conditions, diabetic wounds, and the elderly without booster doses are more vulnerable. Poor hygiene during childbirth also contributes to neonatal tetanus in developing countries. Regions with low vaccination coverage are vulnerable due to lack of awareness, limited healthcare facilities, economic burden, and cultural beliefs against vaccination.

The incubation period of tetanus varies from 3 to 21 days, with an average of around 10 days [4]. *Clostridium tetani* spores enter the body through open wounds when contaminated by environments such as soil or animal feces. Once they enter the body, spores can germinate and multiply in anaerobic conditions, particularly in necrotic tissue. The bacteria produce a neurotoxin called tetanospasmin. Tetanospasmin is released into the bloodstream and travels along motor nerve axons to the central nervous system, where it disrupts the neuronal inhibitory pathways by blocking the release of neurotransmitters like gamma-aminobutyric acid and glycine. These neurotransmitters are essential for muscle relaxation. This blockade causes increased muscle excitability, resulting in the characteristic muscle spasms in tetanus. The common clinical manifestations of tetanus are trismus, opisthotonus, risus sardonicus, dysphagia, painful muscle spasms, and autonomic dysfunction. Sometimes, tetanus can cause life-threatening respiratory failure due to respiratory muscle involvement. Diagnosis of tetanus is primarily clinical. While tetanus typically occurs after a recent wound, rare cases occur without any identifiable entry site, complicating the clinical diagnosis. This case report is unique, as it presents a 68-year-old man diagnosed with tetanus despite the absence of an identifiable wound or trauma.

## Case presentation

A 68-year-old male security officer presented with a three-day history of progressive muscle stiffness and painful spasms. He initially noticed difficulty in opening his mouth, which gradually worsened, followed by stiffness in the muscles of his neck, back, and legs. Over the following days, he developed painful muscle spasms that worsened. Upon further questioning, he denied any history of recent trauma, visible wounds, punctures, injections, or surgeries, and had not engaged in any activities that might predispose them to such injuries (e.g., gardening, handling soil). He was unsure about the last tetanus immunization.

**Figure 1:**
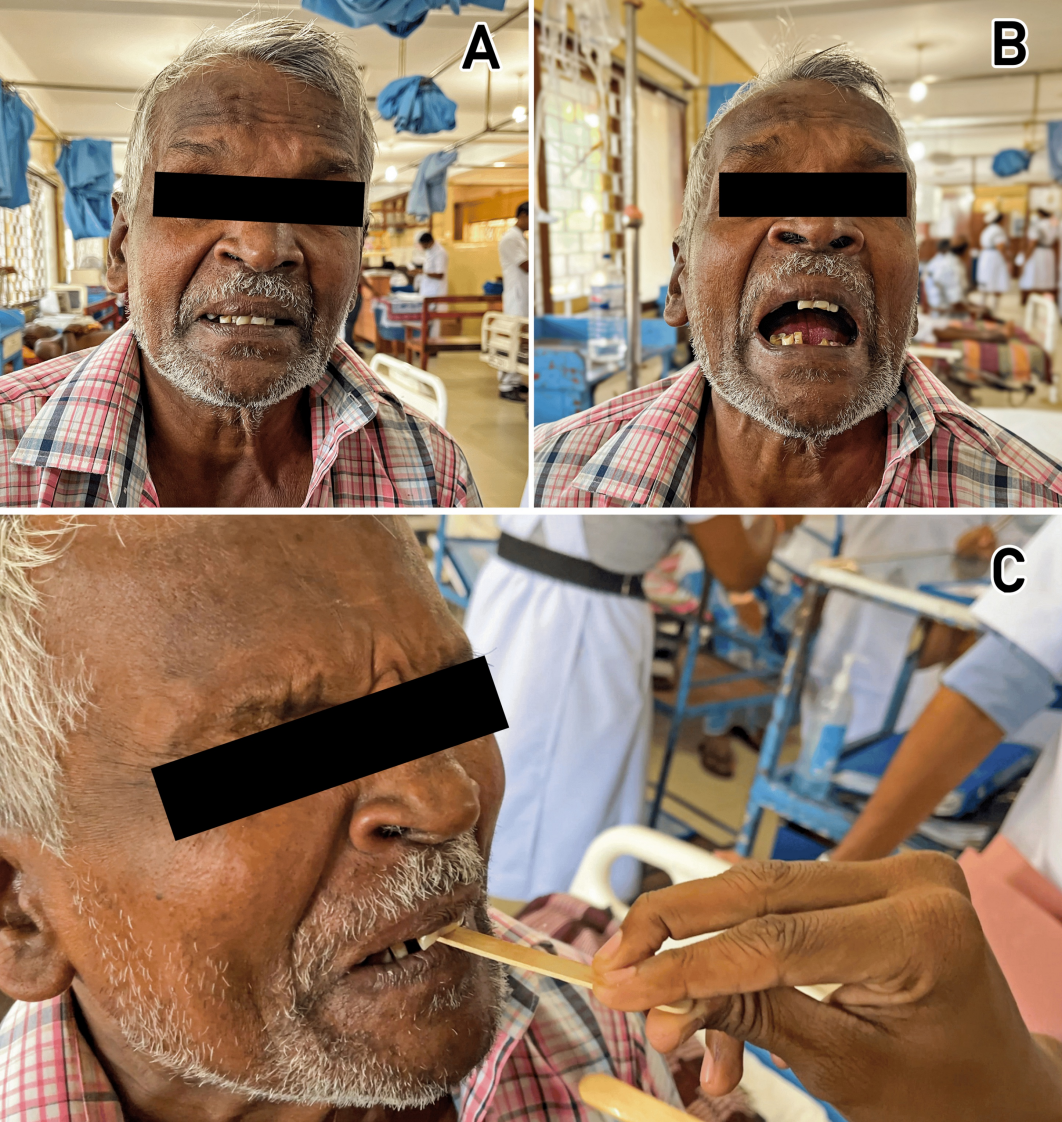
(A) Classic risus sardonicus, a prolonged facial muscle spasm causing an unnatural smile; (B) Trismus, characterized by restricted mouth opening due to spasm of the muscles of mastication; (C) Positive spatula test, showing involuntary biting of the spatula due to reflex spasm of the masseter muscles

On physical examination, the patient was alert and oriented but in noticeable discomfort, with prominent muscle rigidity. The classic "sardonic smile" (risus sardonicus) was noted (Figure 1A), along with trismus, a condition of the restricted opening of the mouth (Figure 1B). He was hemodynamically stable, with blood pressure of 130/86 mmHg, heart rate of 84 bpm, and respiratory rate of 18 breaths per minute. Spasms triggered by external stimulation were minimal. There was no evidence of autonomic instability such as erratic heart rate or blood pressure fluctuations. No wounds or signs of infection were found on a thorough examination of the skin and soft tissues. During the clinical evaluation, the spatula test was performed as part of the bedside assessment for tetanus. In this case, the patient exhibited reflex spasms of the masseter muscles, biting down on the spatula, consistent with a positive test result (Figure 1C).

Laboratory tests, including full blood count, serum electrolytes, serum creatinine, liver function tests, and inflammatory markers, were within normal limits and are summarized in Table 1. Magnetic resonance imaging of the brain hemisphere and brainstem was done to rule out other causes of muscle stiffness but did not show any significant abnormalities. Despite extensive efforts to locate an entry point for *Clostridium tetani* spores, none was found, and tetanus was diagnosed based purely on clinical features with a positive spatula test.

**Table 1:** Summary of the laboratory test results of the patient

Laboratory test	Result	Normal range
White cell count	6.91 × 10^9^/L	4.0-10.0 × 10^9^/L
Haemoglobin	13.0 g/dL	12-15 g/dL
Platelets	195 × 10^9^/L	150-400 × 10^9^/L
C-reactive protein	2.1 mg/L	0-3 mg/L
Erythrocyte sedimentation rate	14 mm/h	Less than 20 mm/h
Aspartate transferase	35 U/L	15-37 U/L
Alanine aminotransferase	50 U/L	16-63 U/L
Albumin	36 g/L	34-50 g/L
Sodium	139 mmol/L	136-145 mmol/L
Potassium	4.0 mmol/L	3.5-5.1 mmol/L
Corrected calcium	2.28 mmol/L	2.1-2.54 mmol/L
Creatinine	65 µmol/L	49-90 µmol/L

The patient was given a 3,000 IU intramuscular injection of tetanus immune globulin (TIG) to neutralize circulating tetanus toxin and prevent further progression of symptoms. Intravenous metronidazole was started to eradicate any possible bacterial source. Muscle relaxants, such as diazepam and baclofen, were administered to relieve the patient's rigidity.

He was monitored in the ward for autonomic instability, a known complication of tetanus. Fortunately, he did not develop any autonomic dysfunction of cardiovascular or respiratory systems. After the 10 days of ward stay, he showed gradual improvement with reduced frequency and intensity of muscle spasms. He was discharged with instructions for follow-up and tetanus vaccination to ensure complete immunization against future infection. At the follow-up visit after two weeks, his symptoms were largely resolved, with marked improvement in muscle rigidity and no further episodes of spasms.

## Discussion

Tetanus is of four types. The most common type is generalized tetanus, which causes widespread spasms throughout the body as in our case. Localized tetanus is limited to the injury site, affecting only the muscles in that area. Cephalic tetanus impacts cranial nerves and can result in facial paralysis, while neonatal tetanus occurs in newborns, often due to poor hygiene during birth. Each type requires prompt treatment to prevent severe complications.

It is well-established that *Clostridium tetani* spores enter the body through open wounds, however, cases without identifiable entry points, as in our case, challenge our understanding of tetanus pathogenesis and warrant a closer look at atypical presentations. *Clostridium tetani* spores may enter the body through microtraumas or unnoticed abrasions that heal quickly and are often overlooked. The development of tetanus without an obvious wound or trauma is a rare but documented phenomenon in the medical literature. A case report by Sivasubramanian G (2020) described a case of generalized tetanus in a 66-year-old landscaper who presented with trismus, dysphagia, and leg cramps. Despite the absence of any visible wounds, which contributed to a delay in diagnosis, the patient was successfully treated once tetanus was clinically diagnosed [5]. Another case report published by Tomoda et al (2018) reported a 68-year-old man presented with fever, neck pain, and severe trismus but no history of trauma or wounds, leading to a clinical diagnosis of tetanus. He was successfully treated with TIG, and intravenous metronidazole, with full recovery in 14 days [6].

Tetanus is primarily a clinical diagnosis, as there are no specific investigations to confirm the diagnosis. The classical clinical features, such as trismus, muscle rigidity, and spasms, and a recent wound form the foundation of the diagnosis [7]. In our case, the absence of a recent wound led to diagnostic challenges, but the positive spatula test during the clinical assessment was crucial to making the diagnosis of tetanus. The spatula test, a simple bedside test, has shown higher sensitivity of up to 94% and specificity of 100% for diagnosing tetanus [8]. The positive test result is indicated by the reflex spasm of the masseters upon touching the posterior pharyngeal wall, as elicited in our case, while the negative test result is characterized by the gag reflex with an attempted expulsion of the spatula. Despite being a useful test, it would sometimes be difficult to perform the spatula test on a patient with lockjaw, as it requires the patient's cooperation in opening their mouth for spatula insertion. "In such cases, an emerging neurophysiological test called the 'masseter inhibitory reflex (MIR)' may be helpful. The masseter muscles are normally reflexively inhibited during biting to prevent excessive force while chewing. However, in tetanus, the hyperexcitability of motor neurons in the central nervous system disrupts this inhibition, eliminating the 'silent period' seen on electromyography (EMG). This loss can help diagnose tetanus, particularly in localized cases like the oromandibular form, where tests like the spatula test may be impractical and conditions like dental abscess do not involve similar neuronal hyperexcitability. The procedure of MIR involves asking the patient to gently clench their teeth while a sterile needle is inserted into one of the masseter muscles. The EMG is set to a gain of 200 μV with a 50 ms sweep speed. A light tap on the chin with a tendon hammer, similar to a jaw jerk test, should normally result in a silent phase on the EMG, indicating masseter muscle reflex inhibition. In conditions like tetanus, where there is central neuronal hyperexcitability, this silent phase is lost [9].

The major objectives of tetanus treatment involve neutralizing the circulating tetanospasmin toxin and controlling spasms. TIG neutralizes circulating tetanospasmin toxin that has not yet bound to nerve endings. However, once the toxin has attached to nerve tissues, TIG is ineffective in reversing its effects. A single dose of 500 IU of TIG is recommended by medical experts for treating tetanus, as it seems to be just as effective as larger doses, ranging from 3,000 to 6,000 IU, despite uncertainty about the ideal dose [10]. In our case, we administered 3,000 IU of TIG intramuscularly. In addition to TIG, antibiotics should be administered to eliminate any local bacterial source. Metronidazole is the preferred antibiotic of choice in tetanus, which was used in our case [11]. Muscle relaxants, such as diazepam and baclofen, are commonly used to manage the symptoms of rigidity and spasms, which was effective in our case [12]. Managing autonomic dysfunction, like blood pressure and heart rate fluctuations, is vital, as severe instability can be fatal. Intensive care monitoring is often needed, but fortunately, our patient had no signs of autonomic dysfunction.

## Conclusions

This case brings the learning point that maintaining a high index of suspicion for tetanus, even in the absence of an identifiable wound. Early recognition remains crucial, as tetanus can present in atypical ways, delaying diagnosis and treatment. The use of clinical diagnostic tools, particularly the spatula test, proved valuable in this scenario, helping establish the diagnosis promptly. Moreover, early and aggressive treatment with TIG, antibiotics, supportive care, and follow-up care significantly improves patient outcomes. This case reinforces the need for continuous clinical awareness of tetanus in both common and uncommon presentations to prevent complications and ensure timely management. This case also emphasizes the importance of preventive measures like vaccination.

## References

[1] (2024). WHO. Tetanus. https://www.who.int/news-room/fact-sheets/detail/tetanus.

[2] Li J, Liu Z, Yu C, Tan K, Gui S, Zhang S, Shen Y (2023). Global epidemiology and burden of tetanus from 1990 to 2019: a systematic analysis for the Global Burden of Disease Study 2019. Int J Infect Dis.

[3] (2024). WHO. SDG Target 3.b. Essential medicines and vaccines. https://www.who.int/data/gho/data/themes/topics/sdg-target-3_b-development-assistance-and-vaccine-coverage.

[4] Hsu SS, Groleau G (2001). Tetanus in the emergency department: a current review. J Emerg Med.

[5] Sivasubramanian G (2020). Generalized tetanus in a landscaper without obvious wounds. IDCases.

[6] Tomoda Y, Kagawa S, Kurata S, Nakatake N, Tanaka K (2018). Tetanus without apparent history of trauma. J Gen Fam Med.

[7] Yen LM, Thwaites CL (2019). Tetanus. Lancet.

[8] Apte NM, Karnad DR (1995). Short report: the spatula test: a simple bedside test to diagnose tetanus. Am J Trop Med Hyg.

[9] Imtiaz H, Hakeem H, Alam A, Kanwar D (2019). Making an objective diagnosis of tetanus—utility of a simple neurophysiological test. BMJ Case Rep.

[10] (2024). CDC. Clinical care of tetanus. https://www.cdc.gov/tetanus/hcp/clinical-care/index.html#:~:text=Medical%20experts%20recommend%20a%20single.

[11] Ahmadsyah I, Salim A (1985). Treatment of tetanus: an open study to compare the efficacy of procaine penicillin and metronidazole. Br Med J (Clin Res Ed).

[12] Ergonul O, Egeli D, Kahyaoglu B, Bahar M, Etienne M, Bleck T (2016). An unexpected tetanus case. Lancet Infect Dis.

